# Media effects on suicide methods: A case study on Hong Kong 1998-2005

**DOI:** 10.1371/journal.pone.0175580

**Published:** 2017-04-12

**Authors:** Qijin Cheng, Feng Chen, Paul S. F. Yip

**Affiliations:** 1 Hong Kong Jockey Club Centre for Suicide Research and Prevention, The University of Hong Kong, Hong Kong, Hong Kong SAR; 2 School of Mathematics and Statistics, The University of New South Wales, Sydney, New South Wales, Australia; 3 Department of Social Work and Social Administration, The University of Hong Kong, Hong Kong, Hong Kong SAR; University of Toronto, CANADA

## Abstract

**Background:**

Previous studies have suggested that mass media’s reports of new suicide methods will increase suicides using the same method. The same pattern seems not to apply to a conventional suicide method, unless it was used by a celebrity.

**Objective:**

1) to examine media effects on both new and non-new suicide methods during 1998 and 2005 in Hong Kong (HK), when a new method by burning charcoal (CB suicide) was spreading in the region. 2) to examine how CB competed with non-CB methods in terms of media coverage and “recruiting” suicidal persons in the socio-economic context.

**Methods:**

A self- and mutual- exciting process model was fitted to the data, adjusting for divorce rate, unemployment rate, and property price index. Breaking the whole period into onset, peak, and post-peak stages, the model was fitted again to examine the differences.

**Results:**

Comparable copycat effects were found on both CB and non-CB suicide news. The only cross-method media effects were found in the onset stage when non-CB suicide news showed suppressing effect on CB suicides. CB suicides reported a significant self-excitation effect. A higher divorce rate and lower property price index were associated with significantly more suicides incidences and more suicide news.

**Conclusions:**

The emerging of CB suicide method did not substitute media coverage of non-CB suicide in HK. Media effects in this case were not limited to new suicide method or celebrity suicide. The effects were further fueled by adverse socio-economic conditions.

## Introduction

Ever since the rise of mass media, such as newspapers, radio, and film, in the 1920s, research on mass media’s effects on people’s attitude, cognition, and behaviors has attracted more and more attention. Contrary to many lay people’s impression, recent meta-analysis studies have noted small to moderate media effects on violent behaviors or suicides [1]. Scholars in the field, hence, stressed that, although mass media are broadcasting to the general population, their effects are conditional [2–3]. Therefore, to specify the conditions that media effects would take place has become a priority in the media studies field [4].

Particularly with suicide, copycat effects were observed under certain conditions, such as when the news was about celebrity suicide [3, 5], and when the news viewers shared a similar profile as the person in the suicide news [6–8]. By contrast, copycat effects were seldom found after ordinary people’s suicide news [9]. The other condition is that when a new suicide method was reported in detail and portrayed as painless or being used by a celebrity, more people would kill themselves using the same method [10–11]. Theoretical explanation of the phenomenon includes that such media reports would facilitate cognitive access to a new method [12–13].

Previous studies have noted that while news reports arise of a new suicide method, news reports of suicides by conventional methods would drop, suggesting a substitution effect [14]. Furthermore, the media effects on copycat suicide were found on a new suicide method but not conventional methods [14–15]. Applying the cognitive access theory, the phenomenon might be explained that the population has already know conventional methods well so conditions will not be significantly changed by a news report.

In summary, previous studies suggest that, mass media effects on suicides by different methods are conditional to whether the method is new. The hypotheses can be summarized that

1. when suicides using a new method are “competing” with those using non-new methods, the new ones would attract more media attention and substitute some media coverage of non-new ones;
2. the substitution effect would further lead to a rapid increase of suicides by the new method; and
3. suicide incidents by non-new methods are not significantly affected by media reports, as long as there is no celebrity suicide being reported.

While examining those hypotheses on the spreading of charcoal burning suicide in Hong Kong, we noted similar but also different patterns. The present paper reports our examination and the findings and discuss why different patterns appeared in our case. Our specific objectives include: 1) to examine the media effects on both new and non-new suicide methods during 1998 and 2005 in Hong Kong, when a new method by burning charcoal in a sealed room (CB suicide) was rapidly spreading in the region; and 2) to examine how CB and non-CB methods competed with each other in terms of media coverage and “recruiting” suicidal persons in the social-economic context.

### The case of charcoal burning suicide in Hong Kong

The study chose the spreading of CB suicide in HK for a case study. In November 1998, a woman’s CB suicide was reported by local newspapers in detail as the first-ever publicized suicide using this method. Within two years, this method became the second most frequently used suicide method in HK and raised serious public health concerns. Previous studies on the spreading trajectory of CB suicide in HK identified 2002~2004 as the peak stage and suggested 1998~2001 to be the “onset” stage and after 2004 to be stabilized post-peak stage [16]. During the years of 1998~2005, there were no celebrity suicides by CB in HK but one by jumping from height which occurred on April 1, 2003. The method was further spread to Taiwan, Japan, Korea, Singapore, and other countries, and has accounted for more than 5000 deaths up to date [17–18]. Therefore, the case is of international interest to inform effective strategies to prevent such epidemics from happening again. However, as far as we know, no quantitative study has been done to examine the media effects in this case in HK. The present study examined the media effects between 1998~2005 and also looked into whether the media effects varied in the onset, peak, and post-peak stages.

## Methods

### Data collection

Official suicide death data between Sept 1, 1998, a couple months before the first CB suicide case was reported by local media, and Dec 31, 2005, when the CB suicide had become the second most used suicide method in HK for four years, were extracted from the Hong Kong Coroner’s Court and Census & Statistics Department. Between 1998 and 2000, the 9^th^ revision of the International Classification of Diseases (ICD-9) was used in HK for coding external causes of death, which have been replaced by ICD-10 since 2001. Following previous studies on suicide problem in HK, we used codes E950-959 (suicide and self-inflicted injury) for ICD-9 and X60-84 (intentional self-harm) for ICD-10 to extract suicide incidence. In addition, we used E952 for ICD-9 and X67 for ICD-10 to identify suicide death by charcoal burning, which are officially indicating death by intentional self-poisoning by other gases and vapors. Previous studies found that the vast majority cases in this code are caused by burning charcoal [16, 19].

Suicide news published in four major newspapers in HK, namely, Apple Daily, Ming Pao Daily, Oriental Daily, and Singtao Daily, was downloaded from WiseNews database. We first used suicide-related keywords, such as “suicide” and various suicide methods, to search the four newspapers’ archives during Sept 1, 1998 and Dec 31, 2005 in the dataset. The downloaded articles were further reviewed by three trained coders to exclude those articles that are not news report of HK resident’s suicide death. However, the WiseNews dataset includes neither Oriental Daily before Jan 1, 2000 nor Apple Daily before Jan 1, 1999. Therefore, we manually reviewed these two newspapers archives at a library to extract suicide news in according periods. Each of the suicide news articles was classified by whether or not the suicide death was reported as using the charcoal burning method.

Although the present study focuses on media effects, we are fully aware that suicide is caused by complex reasons. Therefore, the study took into consideration of some major socio-economic factors. Particularly, three socio-economic factors were often found to be related to suicide: divorce, unemployment, and property price. Previous reviews noted that divorce and unemployment are risk factors for suicide internationally and in HK locally [20–22], which warrant us to take these two factors into consideration in the present study. In addition, it is widely believed by the HK public that numerous suicides were triggered by the negative equity issues, which referred to the sharp drop of private property price that was triggered by the financial crisis in 1997 and lasted till 2003 after the Severe Acute Respiratory Syndrome (SARS) attacked HK [23]. Therefore, we included the private property price index in the study to examine whether such assumption is empirically supported. We extracted daily property private domestic-price index [24], monthly unemployment rate, and annually crude divorce rate from the Hong Kong Census and Statistics Department.

### Data analysis

1478 (19.8%) suicide deaths were given not a precise death date but an estimated period, usually a few days up to a week. The multiple imputation method [25] was adopted to assign an exact date to those cases, where the case date was assumed to be uniformly distributed in the estimated period. The imputation was done a total of 10 times. The following analysis was performed on each imputed dataset. The analyses were then combined to produce a final analysis using Rubin’s rule [25].

The time-varying suicide rates by CB and non-CB methods and the reporting intensities by different newspapers on CB suicides and non-CB suicides were estimated using a nonparametric intensity estimator [26–28]. The analysis aimed to examine any temporal patterns of the suicide rates and reporting intensities and any relationship among those patterns. To formally model the counts of suicide cases by CB and other methods and news articles of CB and other suicide news, we initially performed Poisson and negative binomial regression analyses assuming the four time-series of counts are conditionally independent, with their means depending linearly on the three contextual variables.

The choice of Poisson or negative binomial regression depended on whether there is over-dispersion according to the mean-variance relationship. The PIT (Probability Integral Transform) residuals [29–30] were investigated to assess goodness-of-fit and serial correlation. Since our exploration found the lack of fit and serial correlation, we re-analyzed the data by fitting a self- (and mutual-) exciting process model, which extend the Poisson and negative binomial regression models by allowing the mean rate of any specific type of events (CB suicide, other suicide, CB news, or other news) to depend on the past counts of events of the same or different types, in addition to the contextual variables. The average effects of a certain type event on future event rates of the same or different types are time varying and are each described by a function called the excitation function. The estimated model parameters then allowed us to infer the dynamics between different types of the potential effect while contextual variables are controlled for (for more details, see Text A in S1 File—The Self- And Mutually-Exciting Process Model). The finite order autoregressive Poisson or negative binomial regression model used in previous studies [27, 31] was not used in the present study, because those models suffer from stability issues when the time series to be fitted is long [32], which is the case in the present study.

The self-exciting process model was also fitted to the data from three stages as defined by Chen et al. [16]: “onset” period- 1998 to 2001, “peak” period- 2002~2004, and “post peak” period- 2005.

## Results

**Fig 1:**
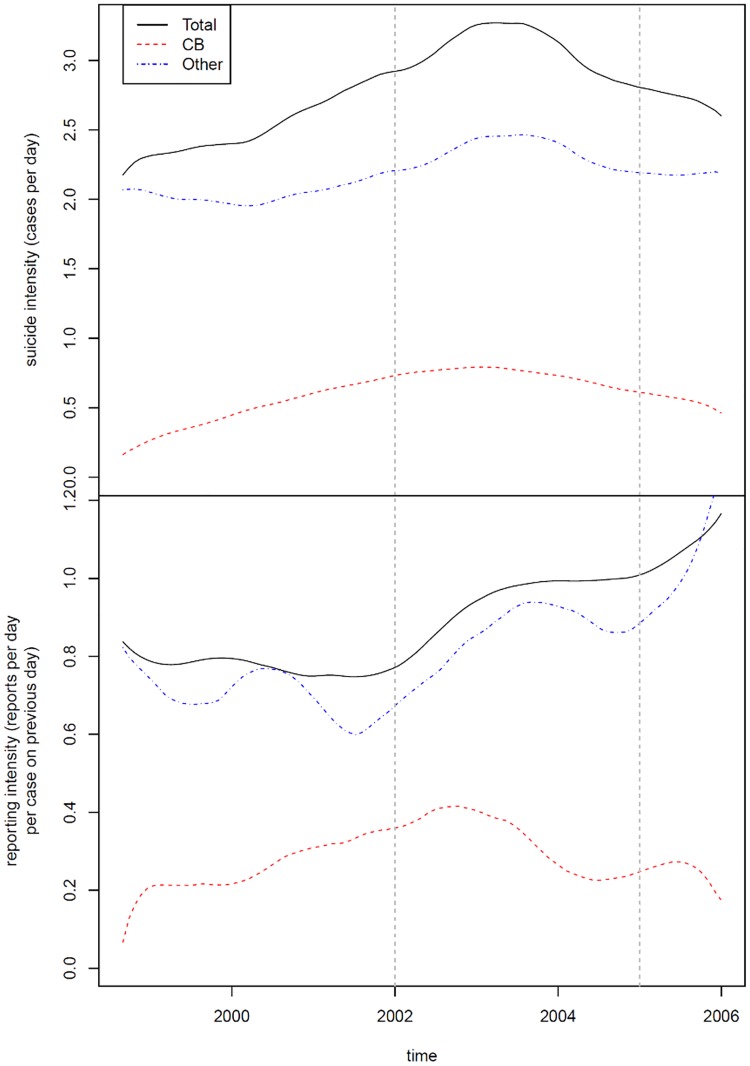
Overall trends of suicide rates and suicide news reporting intensities in 1998–2005.

During the study period, a total of 6958 suicide deaths occurred, of which 19.8% were CB suicides. Meanwhile, 6308 suicide news articles were published, of which 23.1% were CB suicide news. The overall trends of suicide rates and suicide news reporting intensities during the study period are illustrated in Fig 1. As shown in the lower panel of Fig 1, the CB suicide news reporting intensity rapidly went up in the onset stage, whereas the non-CB reporting intensity showed fluctuations. In the peak stage, CB suicide news reporting intensity reached a peak in 2003 and then started dropping, and the downward trend continued to the post-peak stage. Meanwhile, non-CB suicide news reporting intensity was not substituted by CB suicide news but remained in a general upward trend in the peak and post-peak stages. Throughout the whole period, non-CB suicide news reporting intensity constantly outperformed CB suicide news.

In terms of suicide intensity, as shown in the upper panel of Fig 1, there seems to be a decreasing of non-CB suicides associated with an increasing of CB suicide in the onset stage. However, in the peak and post-peak stages, both CB and non-CB suicides showed a similar belt curve, although non-CB suicide stayed at the peak for a longer period in 2003–2004.

**Fig 2:**
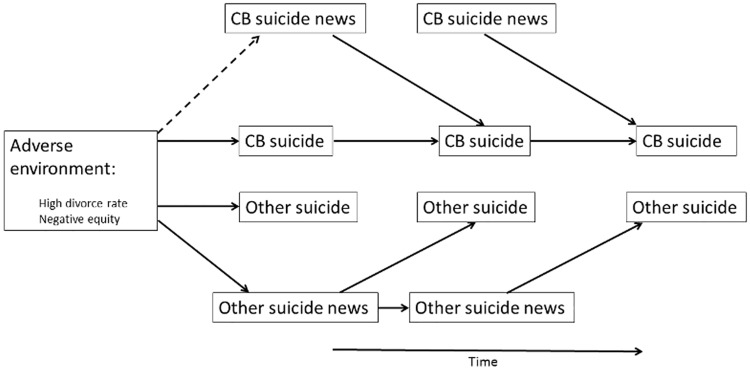
Method-specific media effects in Hong Kong (1998~2005). Note: dash line indicates that only negative equity but not high divorce rate showed significant effects.

The self- and mutually-exciting process models were found to fit well with the data set, as indicated by the diagnostic plots (Text B in S1 File—Histograms and ACF Plots). The results of fitting the model to the whole study period and separated into three stages are presented in Table 1. To visualize the relationships, the results of the whole study period are illustrated in Fig 2

**Table 1:** Estimates of the parameters of the self- and mutual exciting process model fitted to data in whole study period and three stages.

Effects on		Whole period	Onset Stage (1998–2001)	Peak Stage (2002–2004)	Post-peak Stage (Post 2004)
**CB suicide**	(intercept)	1.354 (0.096) ***	1.287 (0.165)***	1.371 (0.142)***	1.278 (0.291)***
	Unemployment rate	-0.023 (0.015)	-0.018 (0.020)	-0.015 (0.023)	-0.003 (0.045)
	Divorce rate	0.186 (0.055) ***	0.159 (0.095)	0.166 (0.071)*	0.152 (0.110)
	Property Price Index	-0.013 (0.001) ***	-0.012 (0.002)***	-0.013 (0.002)***	-0.011 (0.003)**
**Other suicide**	(intercept)	1.843 (0.240) ***	1.792 (0.198)***	1.926 (0.229)***	1.685 (0.295)***
	Unemployment rate	0.041 (0.029)	0.030 (0.042)	0.054 (0.037)	-0.017 (0.062)
	Divorce rate	0.302 (0.123) *	0.316 (0.175)	0.314 (0.146)*	0.278 (0.209)
	Property Price Index	-0.008 (0.003) **	-0.008 (0.004)	-0.011 (0.003)**	-0.008 (0.005)
**CB news**	(intercept)	0.882 (0.257) ***	0.681 (0.279)	0.751 (0.329)*	0.807 (0.412)
	Unemployment rate	0.004 (0.013)	0.025 (0.038)	-0.007 (0.025)	-0.045 (0.082)
	Divorce rate	-0.159 (0.097)	-0.226 (0.140)	-0.123 (0.110)	-0.091 (0.169)
	Property Price Index	-0.005 (0.002) **	-0.002 (0.004)	-0.004 (0.003)	-0.003 (0.007)
**Other news**	(intercept)	-0.666 (0.194) ***	-0.634 (0.361)	-0.587 (0.298)*	-0.820 (0.553)
	Unemployment rate	0.044 (0.026)	-0.025 (0.074)	0.044 (0.042)	-0.067 (0.113)
	Divorce rate	0.865 (0.145) ***	0.810 (0.235)***	0.758 (0.186)***	1.065 (0.305)***
	Property Price Index	-0.010 (0.002) ***	-0.005 (0.006)	-0.012 (0.005)*	-0.006 (0.005)
**Self- and mutual-excitation effects on**					
**CB suicide**	CB suicide	0.122 (0.038) **	0.190 (0.073)**	0.120 (0.038)**	-0.44 (0.148)
	Other suicide	0.060 (0.065)	-0.062 (0.140)	0.207 (0.128)	0.059 (0.136)
	CB news	0.562 (0.063) ***	0.621 (0.072)***	0.664 (0.086)***	0.513 (0.138)***
	Other news	-0.160 (0.116)	-0.353 (0.176)*	-0.008 (0.177)	-0.250 (0.388)
**Other suicide**	CB suicide	-0.003 (0.015)	-0.001 (0.018)	-0.014 (0.025)	0.034 (0.039)
	Other suicide	0.044 (0.032)	0.028 (0.053)	0.045 (0.049)	0.259 (0.138)
	CB news	-0.005 (0.021)	-0.006 (0.023)	-0.014 (0.039)	0.064 (0.066)
	Other news	0.504 (0.035) ***	0.485 (0.045)***	0.551 (0.068)***	0.737 (0.089)***
**CB news**	CB suicide	-0.007 (0.021)	0.006 (0.040)	-0.002 (0.054)	0.006 (0.076)
	Other suicide	0.008 (0.042)	0.091 (0.076)	-0.079 (0.042)	0.016 (0.109)
	CB news	0.067 (0.038)	0.068 (0.049)	0.090 (0.052)	-0.008 (0.068)
	Other news	0.110 (0.071)	0.153 (0.083)	0.121 (0.083)	0.201 (0.258)
**Other news**	CB suicide	-0.007 (0.008)	-0.005 (0.013)	-0.004 (0.013)	-0.018 (0.016)
	Other suicide	0.010 (0.022)	0.040 (0.030)	-0.031 (0.027)	0.061 (0.086)
	CB news	-0.005 (0.011)	-0.012 (0.014)	0.011 (0.016)	-0.021 (0.022)
	Other news	0.109 (0.028) ***	0.129 (0.045)**	0.139 (0.052)**	0.040 (0.051)
**Shape parameter γ of the excitation function**					
**CB suicide**	CB suicide	1.383 (0.625)	2.559 (1.217)	0.100 (0.324)	1.184 (1.725)
	Other suicide	0.890 (1.119)	1.407 (1.754)	2.070 (1.550)	0.684 (0.881)
	CB news	0.728 (0.191)	0.293 (0.150)	1.464 (0.308)	0.584 (0.351)
	Other news	1.841 (1.372)	4.999 (2.932)	1.415 (2.820)	2.640 (2.590)
**Other suicide**	CB suicide	0.499 (1.137)	0.212 (0.496)	0.613 (1.031)	0.345 (0.582)
	Other suicide	0.743 (1.094)	0.834 (1.194)	1.185 (1.922)	1.278 (1.065)
	CB news	1.931 (1.883)	1.878 (2.105)	1.951 (2.166)	2.410 (1.851)
	Other news	0.077 (0.088)	0.062 (0.096)	0.322 (0.164)	0.089 (0.148)
**CB news**	CB suicide	1.046 (1.511)	1.499 (2.421)	1.126 (2.237)	1.414 (1.634)
	Other suicide	1.167 (1.569)	1.518 (1.843)	0.380 (0.652)	1.117 (1.250)
	CB news	1.205 (0.882)	1.454 (1.019)	0.536 (0.615)	1.008 (1.273)
	Other news	1.435 (1.079)	1.644 (0.785)	1.049 (0.864)	1.821 (1.684)
**Other news**	CB suicide	0.078 (0.380)	0.251 (0.507)	0.202 (0.645)	0.003 (0.227)
	Other suicide	0.554 (1.047)	0.353 (0.485)	0.642 (0.958)	1.353 (1.827)
	CB news	0.520 (0.984)	0.603 (1.160)	0.478 (0.774)	0.510 (0.975)
	Other news	0.698 (0.457)	1.299 (0.542)	0.811 (0.542)	0.356 (0.521)
**Dispersion parameter of the negative binomial distributions**					
**CB news**		0.279 (0.021)	0.252 (0.027)	0.342 (0.037)	0.264 (0.053)
**Other news**		1.311 (0.074)	1.131 (0.098)	1.409 (0.122)	1.562 (0.227)

Note:

*p<0.05,

**p<0.01,

***p = <0.001

In the whole period, suicide news showed significant effects on following suicide incidence in a method-specific manner. On average, one CB suicide news article excited 0.562 CB suicide sequentially, whereas one other suicide news article excited 0.504 other suicide. In addition, every CB suicide, on average, excited 0.122 CB suicide sequentially, whereas other suicide did not show such self-excitation. There was neither a significant effect of CB suicide on CB suicide news, nor other suicide on other suicide news. Other suicide news showed significant self-excitation effect, as every non-CB suicide news article averagely excited 0.109 consecutive non-CB suicide news article, but such effect was not found in CB suicide news. In addition, there were no significant mutual-excitation effects between CB suicide news and other suicide news, which further confirmed our observation on Fig 1 that CB suicide did not substitute media’s attention to non-CB suicide.

In terms of socio-economic factors, a higher divorce rate and lower property price index showed significant effects on more incidences of CB and other suicides, but the unemployment rate did not show any significant effects on them.

The shape parameters of the above self- or mutual-excitation effects around or smaller than 1, which indicates that those effects mainly took place in the following one or two days and then quickly diminished.

When breaking down to three stages, the method-specific media effects were found to be consistently significant, although the effects of CB news on CB suicide appeared to be strongest in the peak stage but the effects of other news on other suicide gradually increased along the time. In addition, neither CB suicide nor other suicides showed any significant effects on following suicide news reporting across the three stages.

Different from the observations on the overall period, in the onset stage, every other suicide news, on average, suppressed 0.353 CB suicide sequentially, and the effect is statistically significant. In addition, the self-excitation effect of other suicide news remained significant in Stages 1 and 2, but not Stage 3.

In terms of socio-economic factors, a higher divorce rate and lower property price index showed significant influences on CB suicide, other suicide, and other suicide news in Stage 2, whereas Stages 1 and 3 only showed significant influences of lower property prices on CB suicide and of higher divorce rates on other suicide news.

## Discussion

To the best of our knowledge, this is the first systematic examination on the media effects on the CB suicide epidemic in the HK context. It is also the first-of-its kind study using the advanced self- and mutual- exciting process model to examine the relationships between suicide incidence and suicide news reporting. The results provide new insights into conditional media effects on suicide.

The results support the idea that mass media facilitated the spreading of CB suicide method in HK, which is consistent with previous findings on CB suicide in Taiwan [14]. However, different from previous research findings, the same media effects were also found on non-CB suicides. More interestingly, the media effects on CB suicide appeared to be strongest in the peak stage and then diminished after the peak, but the media effects on non-CB suicide gradually increased along the time.

It is of note that there was no other new suicide method that emerged in HK during the study period. There was only one celebrity suicide, which occurred in 2003 and was found to be associated with a short-term increase of jumping suicide in males of a similar age as the celebrity [5, 33]. However, our results demonstrated that the media effects of non-CB suicide have persisted throughout the five plus years, with the majority of publicized suicides being ordinary people. Moreover, in the onset stage, non-CB suicide news even showed suppressed effects on CB suicide incidence, although the magnitude was not as high as its fueling effects on non-CB suicide.

The significant media effects on suicides using both new and non-new methods, with non-new methods even stronger, might be related with HK media’s particular interests in suicide news. As shown in our results, suicide news reporting intensity in HK remained at 0.8 or more article per case per day throughout the whole study period. When breaking into three stages, CB suicide news reached the highest intensity in the peak stage and then dropped down, whereas non-CB suicide news maintained upward trend and always attracted more media coverage than CB suicide news. The emerging of a new suicide method appeared to have enlarged suicide news coverage capacity instead of substituting non-CB suicide news. HK newspapers’ insatiable appetite for suicide news deserves future cultural and sociological studies. In addition, it is noteworthy that the most popular suicide method in HK is jumping from height, regardless of the onset of CB suicide [34]. Previous studies have noted that jumping suicide tended to receive more media coverage and the news showed more significant effects on sequential suicide incidents [35–36].

Although we focused on the quantity of suicide news reporting in the present study, it is of note that previous studies have observed that, around the same study period, Hong Kong newspapers often reported suicide news with details of personal information, incident location, suicide method, and published photos or graphics frequently [37, 38]. The sensational reporting style might be related to the significant effects of suicide reporting intensity in Hong Kong, regardless of whether the deceased was a celebrity or ordinary person, the suicide method was novel or common.

As previous studies have found that financial crisis and divorce are risk factors for suicide, our study further demonstrated that they are also risk factors for suicide news reporting in HK. Our results support the public perception that the negative equity problem was related with suicide in HK, and that the media coverage interest was reflecting and reinforcing such a perception. In particular, the property price index consistently showed significantly negative effects on CB suicide across the three stages, but its effects on non-CB suicide was only significant in the peak stage. It seems that CB suicides were more influenced by economic context, which is consistent with previous findings that people died by CB suicide were more likely to have unmanageable debt than those died by other suicide method [39]. When the economy in HK gradually recovered after 2003, both CB suicide incidents and CB suicide news reporting decreased. Meanwhile, non-CB suicide news reporting intensity remained increasing. The results warrant more studies on whether there are stereotyped linkages between non-CB suicides and other socio-economic factors, how those stereotypes were formed, and how we can possibly break the stereotypes.

It is of note that we only included three contextual factors in the study, whereas there might be other demographic (e.g. gender and age), social, and economic factors that have influenced suicide incidence. Nonetheless, the diagnostic plots showed that our models are well fitted to the data and justified the validity of our results. The other limitation of the study is that we only focused on print media but not other mass media, such as television or radio news. Nonetheless, we have observed that Hong Kong TV and radio channels rarely report suicide news unless the deceased was a celebrity or part of a suspected suicide cluster. Therefore, for the present study topic, the impacts of TV or radio news might be minor. The difference is because the broadcasting business in Hong Kong is under much stricter regulations than print press. The study is also limited by not analyzing content characteristics of the suicide news. Content analysis should be able to forward our knowledge of conditional media effects. However, going through the 6000+ news articles and identifying and extracting relevant features of the text requires substantial extra work, and therefore we leave it to a future study.

### Implications

As found in previous qualitative studies, media professionals were often skeptical with the copycat effects of suicide and requested academia to provide stronger and localized evidence [40]. The present study provides empirical evidence of mass media’s crucial role in suicide prevention in a society like HK. It can equip suicide prevention professionals to raise local media professional’s awareness of their responsibilities in suicide prevention and persuade them to improve their practices.

In addition, it demonstrates that, when a new suicide method is emerging in a society that is facing socio-economic adversity and has sensational local media, intervention strategies should not be limited to constraining cognitive access to the new method. Instead, we need to eliminate mass media’s overall coverage of suicide news, and work together with media professionals to break the stereotype that more suicide news would occur responding to adverse socio-economic conditions. Alternatively, mass media may cover more positive cases of people who overcame their life difficulties, which were found to be protective for suicide [35].

## Conclusion

The spreading of CB suicide has taught us a painful lesson. The present study shows that, in a society like HK, media interests were of suicide rather than a particular suicide method. In this context, media effects on suicide were not limited to suicides using a new method or celebrity suicide. In addition, not only suicide incidence but also media interests in suicide appeared to be influenced by adverse socio-economic conditions, where the latter might have further worsened the former association.

In summary, suicide prevention is an everyday battle and requires close cooperation of suicide prevention professionals, media professionals, and policy makers. Improving the social economic environment is important for protecting the population from suicide risk, whereas strategically and constructively changing media reporting practices is certainly a powerful weapon in defending lives against suicide.

## Supporting information

S1 File Text A: The Self- and Mutual-Exciting Process Model; Text B: Histograms and ACF Plots.
(DOCX)
