# Supplementary material for: Media effects on suicide methods: A case study on Hong Kong 1998-2005
Source: PLoS One. 2017 Apr 12;12(4):e0175580. doi: 10.1371/journal.pone.0175580 (PMC5389840; doi:10.1371/journal.pone.0175580)
Supplement: S1 File — Text A: The Self- and Mutual-Exciting Process Model; Text B: Histograms and ACF Plots. (DOCX) [file pone.0175580.s001.docx]

**Text A - The Self- and Mutually-Exciting Process Model**

Let $\mathcal{y}_{t}^{i}, i=1, . . . , d$, denote the number of events of different type $i, i=1,. . . , d$ at time$t, t=1, . . . , T$. In the Hong Kong suicide incidence and newspaper reporting data to be considered below, $d=4,$ type 1 event indicates charcoal burning suicide, type 2 indicates newspaper reporting of charcoal burning suicide, type 3 indicates non-charcoal burning suicide, type 4 event indicates newspaper reporting of non-charcoal burning suicide cases. Assume that given $\mathcal{F}_{t-}$, the information available prior to time *t*, the counts $\mathcal{y}_{t}^{i},i=1, . . . , d$, are independently distributed according to Poisson or negative binomial distributions with means $\lambda^{i}\left( t \right), i=1, . . . , d$ respectively. To explore the potential self- and mutual- excitation/inhibition effects between events of the same or different types, we assume the following structure for the conditional mean number of events $\lambda^{i}$,

$$\lambda^{i}\left( t \right)=\left( {x(t)}^{⊺}\beta^{i}+\sum_{k=1}^{d} \sum_{j=1}^{t-1} \alpha_{k}^{i}\mathcal{y}_{j}^{k}g(t-j; \gamma_{k}^{i}) \right)^{+}, (1)$$

where $\mathcal{x}$(*t*) is the *p*-vector of the values of the external variables at time *t*, and ${(\cdot)}^{+}= \cdot\vee0$denotes the positive part of $\cdot$. In analyzing the Hong Kong suicide data, the external variables we consider include the social-economic status variables, such as the unemployment rate, the divorce rate, and the property price index. The constant $1$ is also included to permit an intercept term in the model. The parameter $\beta^{i}$ is the *p*-vector of regression coefficients measuring the influence of external variables on the event rate of type *i* events, $\alpha_{k}^{i}$ measures the excitation/inhibition effect of a type-*k* event on the event rate of type-*i* events, and the $g(t, \gamma_{k}^{i}$), normalized such that $\sum_{t=1}^{\infty} g\left( t, \gamma\right)=1$ specify the distribution of the excitation/inhibition effect over time. We refer to the function $g\left( \cdot, \gamma\right)$ as the excitation function, which has different shapes depending on the value of the parameter $\gamma$. In the discrete time mutually exciting process model, common choices for the excitation functions are probability mass functions supported by the positive integers, such as the truncated Poisson probability function (Porter and White, 2012):

$$g\left( t,\gamma\right)=\gamma^{t}\mathcal{e}^{-\gamma}\left\{ t!(1-\mathcal{e}^{-\gamma})\} \right.^{-1}, t=1, . . . ,$$

which for $\gamma\leq2$, is a decreasing function in $t \in\{1, 2, . . . \}$, and is a unimodal function of *t* with a peak around $\gamma$ for $\gamma>2$. The model (1) is a continuous-time version of the mutually exciting process of Hawkes (1971). A univariate version of this model has been used in the modelling of terrorist activities by Porter and White (2012).

Estimation and inference of the model can be based on the familiar maximum likelihood method. Depending on the form of the excitation function, the likelihood surface could be rather flat along some dimensions of the parameter space resulting in the Hessian matrix being close to sigular or near the maximum, which leads to difficulty in estimating the variance of the ML estimator by inverting the observed information. To overcome this issue, we use a percentile bootstrap approach (Davison and Hinkley, 1997). After obtaining the MLE of the parameters, we simulated a set of B = 200 bootstrap datasets from the self- and mutual-exciting process model with the parameters fixed at their estimates by the ML method, and then re-estimate the model based on each of the simulated bootstrap data sets using the ML method, to obtain a bootstrap sample of the MLE. The percentile bootstrap confidence intervals for each dimension of the parameter vector is then constructed, where the lower and upper 2.5 percentiles are taken as the lower and upper limits of the 95% confidence interval. Significance of each parameter is assessed by whether its bootstrap confidence interval thus obtained contains the hypothesized value when the parameter is assumed non-significant.

**Refernces**

Davison, A. C. and Hinkley, D. V. (1997). *Bootstrap Methods and their Application.* Cambridge Series on Statistical and Probabilistic Mathematics. Cambridge University Press.

Hawkes, A. G. (1971). Spectra of some self-exciting and mutually exciting point processes. *Biometrika*, 58(1):83-90.

Porter, M. D. and White, G. (2012). Self-exciting hurdle models for terrorist activity. *The Annals of Applied Statistics*, 6(1):106

**Text B - Histograms and ACF plots of the PIT residuals of fitting the self- and mutual-exciting process models to the data of the whole period and the three stages.**

*Note: cb = charcoal burning suicide, ot = other suicide, newscb = charcoal burning suicide news, newsot = other suicide news.*

1. Fitting the models to the data of the whole period.

1. Fitting the models to the data in Stage 1 (1998 - 2002).

1. Fitting the models to the data in Stage 2 (2002-2004).

1. Fitting the models to the data in Stage 3 (2005).
